# Upscaling effects on infectious disease emergence risk emphasize the need for local planning in primary prevention within biodiversity hotspots

**DOI:** 10.1038/s41598-025-21514-4

**Published:** 2025-10-27

**Authors:** Renata L. Muylaert, David A. Wilkinson, Evita Izza Dwiyanti, David T. S. Hayman

**Affiliations:** 1 Sydney School of Veterinary Science, Faculty of Science, The University of Sydney, Sydney, NSW 2050 Australia; 2 UMR ASTRE, CIRAD, INRAE, Université de Montpellier, Plateforme Technologique CYROI, Sainte-Clotilde, Reunion France; 3 Alam Sehat Lestari, Jakarta, Indonesia; 4 Molecular Epidemiology and Public Health Laboratory, Hopkirk Research Institute, Massey University, Palmerston North, New Zealand

**Keywords:** Emerging infectious diseases, Remote sensing, Forest management, Biodiversity, Risk assessment, Conservation, One health, Spatial scale, Ecological modelling, Infectious diseases

## Abstract

Zoonotic risk assessments are increasingly vital in the wake of recent epidemics. The microbial diversity of parasitic organisms correlates with host species richness, with regions of high biodiversity facing elevated risks of emerging zoonotic infections. While habitat loss and fragmentation reduce species diversity, anthropogenic encroachment, particularly in forested areas, amplifies human exposure to novel pathogens. This study integrates host habitat, biodiversity, human encroachment, and population at risk to estimate novel disease emergence and epidemic risk at multiple spatial scales. Using Java, Indonesia, as a case study, we demonstrate that degrading spatial resolution leads to information loss, with optimal resolutions typically below 2000 m, ideally around 500 m when native-resolution processing is unfeasible. Gravity models of epidemic spread highlight Jakarta and West Java as high-risk areas, with varying contributions from surrounding regions. Our spatial analysis underscores the influence of population centers on forest management and agroforestry practices. These findings offer valuable insights for guiding pandemic prevention research and improving pathogen- and driver-based risk monitoring strategies.

**Supplementary Information:**

The online version contains supplementary material available at 10.1038/s41598-025-21514-4.

## Introduction

In recent years, the emergence of novel infectious diseases has posed significant challenges to global public health and biodiversity conservation. Understanding the interplay between biodiversity, fragmentation, and the risk of infectious disease transmission is crucial for effective risk assessment and mitigation strategies. The increase in contact areas among humans and animals^1^ due to anthropogenic change is globally pervasive and thought to increase the risk of novel infectious disease emergence. However, there is a dearth of mechanistic, general models and guidelines regarding spatial scale and domains to investigate pathogen emergence and outbreak risk at the wildlife-human and wildlife-livestock interfaces^2–4^. Studies often focus on mapping drivers and stressors at coarse and global scales^5,6^ without acknowledging the potential effect of spatial scale and units used on estimated outcomes. Moreover, studies frequently evaluate scenarios due to the lack of infection data for large areas^7–9^ and strong effects of infection detection and public health access locally^10^.

A crucial driver of emergence is land use change and human encroachment. To date, infectious disease emergence risk is concentrated in tropical, biodiverse forests^11,12^. Because of that, theoretical models can leverage forest management data and landscape metrics to reveal nuances on risk variation throughout scales and the human-wildlife interface, mapping where there is high ecological opportunity for disease emergence^13^. Spatial models are essential for land use planning and, especially in the last 20 years, integrated methods linking biodiversity and infectious disease emergence risk are available for unraveling patterns at multiple scales^2,14,15^. These approaches support the idea that by managing habitats where biodiversity is high, we can reduce the risk of pathogen emergence—an essential concept in primary pandemic prevention^16^. Pandemic primary prevention involves protecting ecosystems to reduce the risk of zoonotic diseases spilling over to humans. This includes conserving biodiversity, regulating wildlife trade, and managing habitats to minimize human-wildlife interactions. These actions aim to preserve natural barriers that prevent the emergence and spread of infectious diseases.

Mapping pathogen-based and driver-based surveillance targets for epidemic prevention initiatives has been identified as critical priority^17,18^. Consequently, it is desirable to investigate how spillover risk varies across management units and how efforts are allocated for different types of managed forests. This is particularly relevant for designing ecological interventions to prevent spillover, as well as for identifying primary prevention targets and associated costs^19^. This can be done through modelling the spillover risk and inspecting how the risk of spread varies according to different levels of managed land throughout an area of interest. In Indonesia, for example, regencies—which encompass rural areas and cities—serve as spatial management units that approximate the structure of gravity models informed by population centers. With the strengthening of epidemic prevention initiatives in Indonesia^20,21^ and globally^16^, advancing cross-sectoral primary prevention projects emerges as a tangible direction to pursue.

The variation in risk across spatial scales and the impact of resolution on spatial estimates, particularly those dependent on edge density and biodiversity, remain poorly understood. Therefore, in addition to estimating risk, it is crucial to examine how the risk of emergence and spread is influenced by spatial resolution, and considering computational costs associated with the processing of large datasets. This includes assessing the extent of information loss when comparing coarser resolutions, such as 1 km rasters, with finer-grained data that is now available for multiple variables, as detailed as 30 m or less for global land cover^22^ and at least 100 m for human spatial demography^23^ .

This work builds upon risk assessments based on drivers^7^ and spillover pathways coupled with estimates of microbial diversity and a mechanistic view of emergence risk in terrestrial areas^2^. Specifically, we: (1) Identify geographical risk zones by estimating the risk for novel infectious disease emergence (eRIDE); (2) Assess estimated epidemic risk in the region across varying spatial scales, examining the loss of information as we upscale spatial resolution moving from finer scales (population centre-informed spatial domains and pixels) to broader spatial domains (provinces and larger pixels); and (3) Analyze how average risk values differ across landscape management classes, from unmanaged to managed forests, and discuss the implications for developing analysis and spatial planning strategies at different risk levels. We expect that low levels of population at risk (PAR) would be associated with low to average land use cover in less-managed forests, as large settlements are typically located in urban areas, while human encroachment is more likely in managed forest types. By analyzing variations in scale and the relationship between PAR and forest management, we aim to support accessible strategies for spatial planning that consider differing scales and risk levels. This approach will inform primary prevention and One Health actions in protected and conserved areas at risk interfaces, helping to develop management strategies that mitigate infectious disease spread while addressing public health and conservation challenges. We apply this framework to the tropical forests of Java, Indonesia, which are at high risk for the emergence of new pathogens^12^ and are also facing significant threats to biodiversity due to anthropogenic pressures^24^ and high population density^25^.

## Results

We analysed the forest area (dense tree cover) comprising 732,113 fragments in Java, Indonesia. Individual forest fragment areas ranged from 0.1 ha (0.001 km^2^) to 347,815.3 ha (3,478.153 km^2^), with an average patch size of 5.2 ha (0.053 km^2^).

The average population density in Indonesia is 144 people/km^2^ (~ 1.44 people per ha), with a maximum of 30,327 people estimated in a single 100 m cell (1 ha) in Java island.

**Fig. 1:**
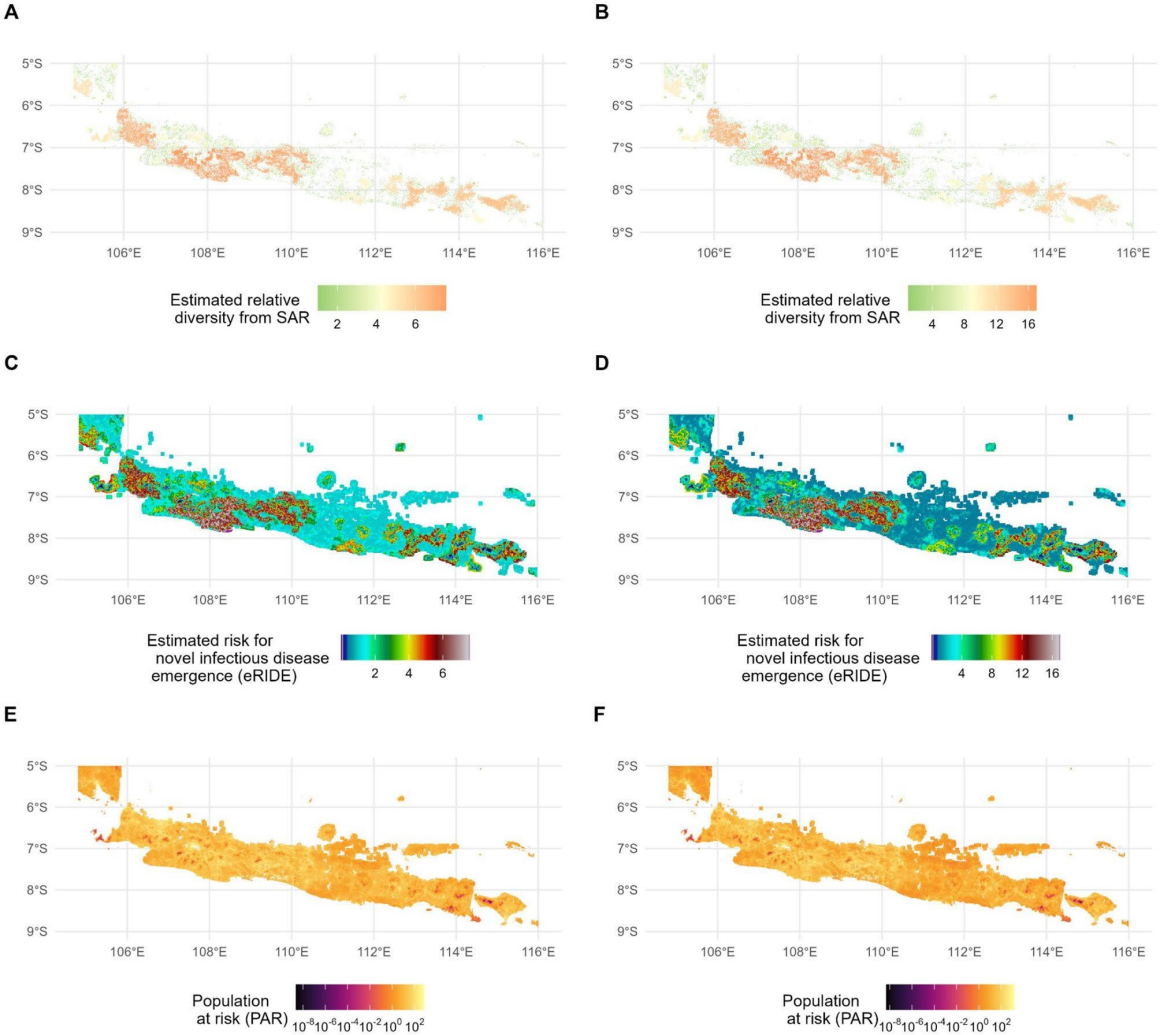
Results for biodiversity, eRIDE, and population at risk (PAR). Results displayed for 500 m resolution. The left panel represents estimates based on z = 0.2, right panel displays estimates based on z = 0.28. Maps were created using R version 4.5.1^26^.

We calculated eRIDE estimates using z-values equal to 0.2 and 0.28 (Fig. 1). Although the magnitude of values differs, their spatial trends and distribution exhibit a similar pattern. Higher z-values will lead to larger mean estimates for biodiversity (and therefore microbial diversity), eRIDE and PAR at 500 m. At z = 0.28, biodiversity was approximately twice as large than at z = 0.2, values for eRIDE 77% higher and values 62% higher for PAR (Figure S1, Table S1). With that, we proceeded with displaying a more conservative estimate using z = 0.2 in the main text.

**Fig. 2:**
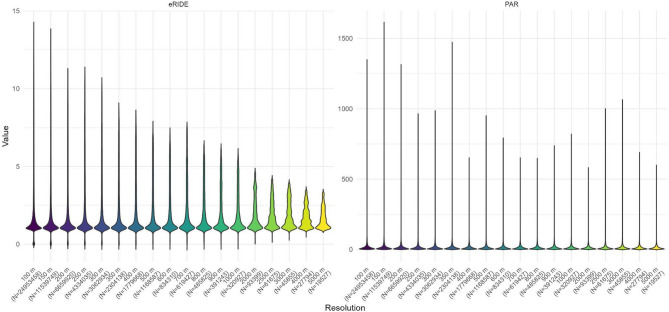
Upscaling effect of estimated risk for novel infectious disease emergence (eRIDE) and human population at risk (PAR) value distribution.

**Fig. 3:**
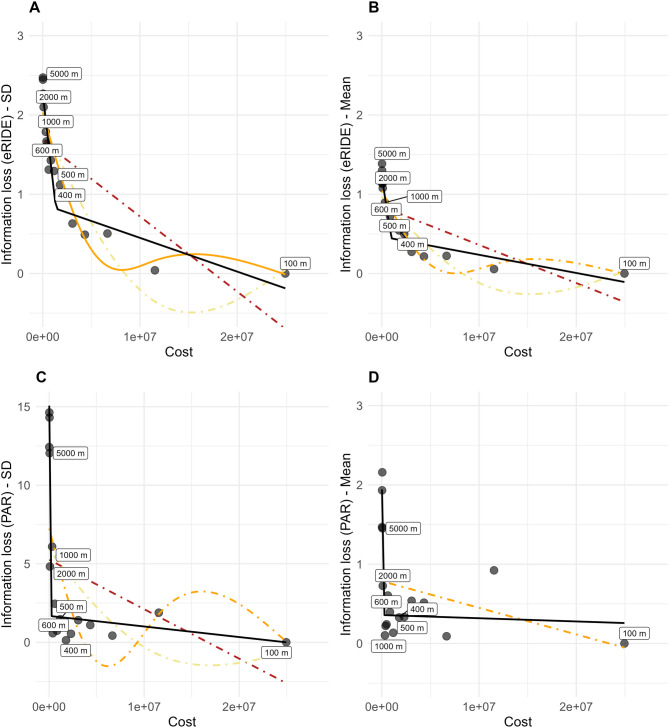
Relationship between resolution upscaling and the risk of novel infectious disease emergence (eRIDE) and population at risk (PAR). (**A**) eRIDE—Standard deviation; (**B**) eRIDE—Mean values (N = 18); (**C**) PAR—Standard deviation; (**D**) PAR—Mean values. Plausible models are shown as continuous lines: orange—GAM (k = 4), a generalized additive model using cubic regression splines with up to four basis functions; black—piecewise model. Information loss was measured as the difference in standard deviation and mean from the reference resolution for each metric.

Regarding the upscaling effect on information loss in eRIDE and PAR, we found that competing models converged for both metrics and differed from a no-effect model, showing that upscaling has clear effects on the value distribution of metrics (Table 1, Fig. 2). Upscaling effect sizes differed between eRIDE and PAR according to plausible models, but both metrics followed a piecewise pattern, with clear breakpoints (Fig. 3). The optimal resolution (the one that minimises information loss with the least amount of cost) revealed by the estimated breakpoint value (*Ψ*) was never coarser than 2000 m when it comes to PAR and below 600 m when it comes to eRIDE (Table 2). Further, adopting a more conservative approach, the working resolution should ideally remain up to or around 500 m when there are no computational limitations, as this was the finest optimal resolution found, minimising information loss and computational costs. The upscale effect for PAR showed a slightly inconsistent decay in information loss in comparison to eRIDE. This type of unstable increase in information loss might be attributed to the high skewness of population data estimates (and large standard deviations) and in part to a discretization effect of neighborhood upscaling for non-integer multiples of the native resolution (150, 250 m, 350, 450 m) when distributing and aggregating values^27^.

**Table 1:** Best supported models to explain resolution effect on eRIDE and PAR based on corrected Akaike Information Criteria and spatial scales (N = 18). Plausible models are in boldface (AICc < 2). df: Degrees of freedom. Weight: weight of evidence.

Metric	Statistical parameter	Model	dAICc	df	Weight
**eRIDE**	**SD**	**Piecewise**	**0**	**5**	**0.982**
eRIDE	SD	gam k = 4	8.1	4.9	0.017
eRIDE	SD	gam k = 3	14.6	4	< 0.001
eRIDE	SD	glm	27.6	3	< 0.001
eRIDE	SD	No-effect	38.7	2	< 0.001
**eRIDE**	**Mean**	**Piecewise**	**0**	**5**	**0.9901**
eRIDE	Mean	gam k = 4	9.3	4.9	0.0095
eRIDE	Mean	gam k = 3	15.7	3.9	< 0.001
eRIDE	Mean	glm	25.8	3	< 0.001
eRIDE	Mean	No-effect	35.2	2	< 0.001
**PAR**	**SD**	**Piecewise**	**0**	**5**	**1**
PAR	SD	gam k = 4	31.3	4.7	< 0.001
PAR	SD	No-effect	31.8	2	< 0.001
PAR	SD	gam k = 3	31.8	3.6	< 0.001
PAR	SD	glm	32.1	3	< 0.001
**PAR**	**Mean**	**Piecewise**	**0**	**5**	**1**
PAR	Mean	No-effect	19.6	2	< 0.001
PAR	Mean	glm	20.6	3	< 0.001
PAR	Mean	gam k = 3	20.6	3	< 0.001
PAR	Mean	gam k = 4	20.6	3	< 0.001

**Table 2:** Breakpoint estimates identify the pixel cost value below which information loss accelerates disproportionately faster. Models considered central measure parameters difference as information loss of two metrics: eRIDE and population at risk.

Metric	Statistical parameter	*Ψ* (cost threshold)	*Ψ* (std error)	Optimal spatial scale range (m)
eRIDE	SD	1,361,292	220,364	400–500
eRIDE	Mean	968,157	162,371	500–600
PAR	SD	137,562	25,507	1000–2000
PAR	Mean	140,259	39,315	1000–2000

**Fig. 4:**
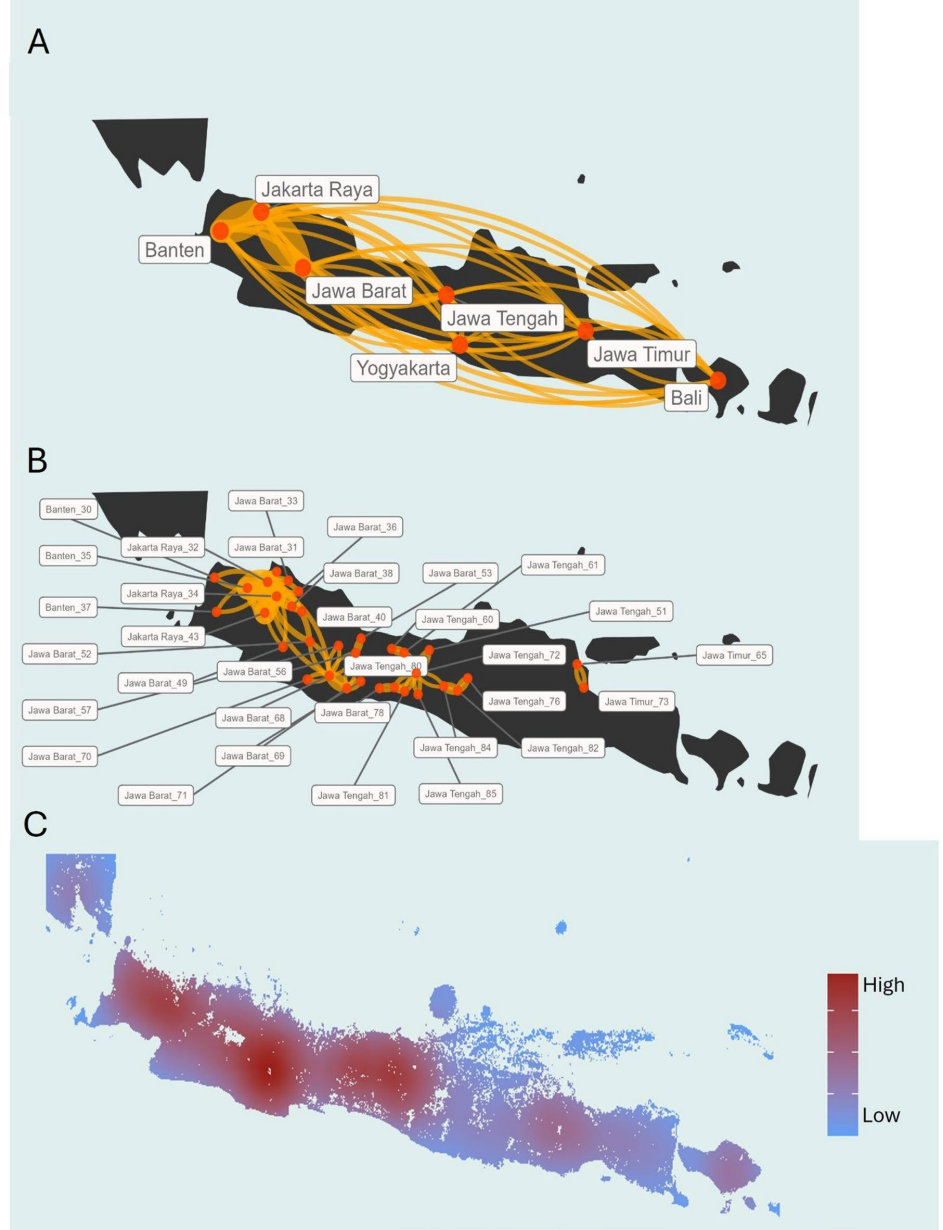
Spatial representation of epidemic risk flowing from provinces in Java. Risk was estimated through a gravity model using: (**A**) Java provinces; (**B**) Highly populated spatial domains (log scale for better representation of links), flows larger than 15 (N = 33) for the Voronoi-driven gravity estimates are displayed. (**C**) Pixel-based received risk based on a 100 km impact zone throughout a gaussian decay. Maps were created using R version 4.5.0^26^.

The gravity models converge into large flows for west Java regardless of spatial units used (Fig. 4). The highest estimated risk values are observed in Jakarta, Jawa Barat and Jawa Tengah (Figs. S4, S5). The greatest contributions to Bali province’s epidemic potential originate from Jawa Timur, while Banten receives most of its risk from Jakarta. Jawa Barat experiences a peak in received risk from Jakarta, followed by Banten, whereas Jawa Tengah receives the largest amount of risk from Jakarta and Yogyakarta. Jawa Timur is primarily influenced by contributions from Jakarta and Jawa Tengah, while Yogyakarta receives a significant portion of its risk from Jawa Tengah, followed by Jakarta.

**Fig. 5:**
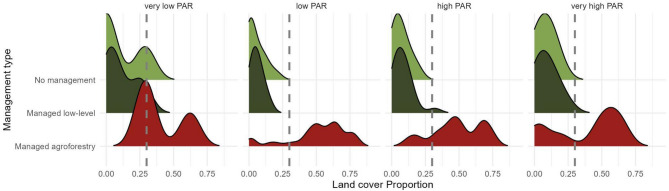
Relationship between land cover of different forest management types across provinces and discretized population at risk estimates. Risk estimates were calculated from the eRIDE model (100 m spatial scale) for high-pop driven gravity model spatial domains. Categories of PAR are based on: Very low = below -1 SD, Low: -1 SD and below mean, High: above the mean, Very high: above + 1 SD. The vertical dashed line marks 30% land cover, a common threshold used in conservation targets^28^.

In terms of forest management and spatial domains, the patterns of sub-regions informed by population centres captured the variation in quantity of managed forest and population at risk, revealing higher values for both Central and West Java (Fig. S6), and largely from Jakarta. The variation of managed agroforestry amount is larger than unmanaged (no management) and low-level managed forest by far (Figs. 5, S7), regardless of the PAR in the region (Fig. S7). Moreover, agroforestry was the predominant land management practice across all regions (Fig. S8). From the data distribution, on a broader, regional scale (Province level), managed agroforests were associated with reduced levels of PAR. However, when local variation is considered (Voronoi-based), unmanaged or minimally managed forests are associated with increased numbers of PAR, but managed agroforests did not display a noticeable change. At the Java island scale (pixel level data), PAR maximums reach similar levels between areas covered by agroforestry and non-managed forests, but areas covered by forests managed at low levels present slightly lower PAR values (Fig. S9).

## Discussion

Upscaling spatial data had a large effect on the information loss for the estimated risk of infectious disease emergence and spread across Java, a biodiversity hotspot. Biodiverse regions, rich in microbial diversity, often serve as reservoirs for zoonotic diseases, where the complex interactions between wildlife, humans, and the environment can increase the risk of disease transmission. However, the variability in spatial resolution and data granularity can obscure patterns and lead to inaccurate risk assessments. Because of that, we found finer resolutions to apply in calculations of epidemic potential. When estimating epidemic potential, we reveal contributions from different provinces to spread, highlighting the role of Jakarta and West Java on risk and land use management. When looking at forest management, a large variation in agriculturally managed forests was found, whereas intact, low-managed forests were most frequently below 30% land cover, a common mark used in conservation targets^28^. That means exposure needs to be minimised through primary prevention actions that manage to reduce the negative effects of human encroachment on biodiversity conservation and human health. Our findings support that the scale for this management should be local and not necessarily based purely on high-level administrative boundaries.

We described how upscaling spatial resolution (finer scales like population centers and from provinces to pixels) affects the accuracy and utility of risk estimates. By upscaling spatial data, we can achieve faster results for estimating disease emergence across different geographical scales from regional to global. However, upscaling results in information loss, especially for data coarser than 2 km resolution that depends on landscape configuration. This is especially important for metrics derived from edges representing the interface of contact where spillover occurs and infectious disease emerges. Our results highlight the importance of considering these factors when integrating the use of finer spatial details to larger spatial scales, which is essential for identifying high-risk areas that may otherwise be overlooked.

The extent to which the area effect on gravity models should be considered is unknown, and to an extent, it is a practical decision taken depending on data and computational capacity provided. Here we have the power to create mechanistic models based on expected biodiversity responses to fragmentation and exposure through different amounts of human encroachment through forest management. Our mechanistic approach can be useful for examining biodiversity and interfaces of risk at scales that minimise information loss from fine grain data, from landscape metrics to risk of spread based on human population. Importantly, the predictive power of spatial models that use microbial diversity as an input is likely to decrease when relying on data with high information loss, as the statistical distribution of the data changes—particularly at resolutions coarser than our estimated threshold values.

Currently, only 12.2% of Indonesia’s terrestrial areas is covered by protected areas (WDPA, 2023), which means that a substantial amount of forest is under little management but not protected. So how things are managed will be very important to determine biodiversity-health outcomes. Our findings highlight the importance of incorporating detailed spatial domains, such as sub-regions informed by population centers, into forest management strategies. The variation in both the quantity of managed forest and population at risk (PAR) is most pronounced in Central and West Java, regions that consistently display higher values for both metrics. This suggests that these areas warrant targeted management interventions to balance biodiversity conservation with human safety when encroaching forests.

There was larger variation in the extent of managed agroforestry compared to non-managed or low-level managed forests. This pattern holds irrespective of the PAR level within a region, indicating that agroforestry management plays a pivotal role in mediating forest resource availability and risk distribution in Java. The greater variability in agroforestry management might reflect differences in local practices, governance, or socio-economic conditions, which deserve further investigation to inform adaptive management policies. Our multi-scale approach underscores the complexity of spatial relationships between forest structure and distribution and human populations. Our insights contribute to a more comprehensive understanding of how managed forest systems are placed in relation to population centres, offering valuable guidance for policymakers aiming to optimize forest sustainability while minimizing epidemic and pandemic risks.

Our workflow can contribute to clearer understanding of how choices in methodology influence estimates of biodiversity and infectious disease risk from theoretical relationships around landscape structure, biodiversity and microbial diversity within that biodiversity. Ultimately, when surpassing the calculated thresholds for information loss, upscaling hinders the ability to develop targeted interventions, prioritize resources, and inform policy decisions that can mitigate the spread of infectious diseases from hotspots. By examining the intersection of infectious disease risk and land use management in tropical forests, we aimed to provide insights into integrated strategies, such as ecological countermeasures, that address health and land use planning challenges simultaneously. We believe that further research on spatial resolution effects is needed to improve spatial planning and advance the application of remote sensing in disease ecology and applied research^29^. This is especially important in areas where local actions may vary widely across healthcare access levels. In the case of Java, when we consider human density and healthcare access, the mode for Indonesia corresponds to low population density close to healthcare^30^. However, this calculation is based on mobility and does not take into consideration equality in healthcare service provision.

### Caveats—scale-dependency, data availability

Population at risk values exhibit anomalous behavior when upscaled to coarser resolutions, warranting further investigation to understand the underlying causes. Latitudinal gradients^31^, mechanisms and drivers of risk can be a multitude of factors that are not patch-based or even visible when looking at general patterns for human demographics. Here, we used Indonesia (Java region) as a case study to understand information loss and resolution of spatial domain when applying mechanistic models for estimating epidemic risk. However, when estimating eRIDE for a large latitudinal gradient, the latitudinal diversity gradient (LDG) may constrain the upper limits for biodiversity and it can be accounted for using our custom functions^32,33^. Because of that, restricted the calculation for the SAR biodiversity component using the observed area of habitat for terrestrial wild birds and mammals and restricted our analysis for Java^34^. In summary, eRIDE workflow includes scaled latitudinal positions for each forest fragment, while occupancy layers for wildlife^35^ can potentially be jointly used to account for variations in macroecological patterns that may exert top-down influences on patch biodiversity across large regions, such as entire continents or global terrestrial areas. Finally, a key limitation of our study is the lack of spatially explicit data on microbial diversity and zoonotic spillover events finely attributed in space. This precludes formal validation using sensitive data that are often biased by disease outbreak detection processes^10^ and reflects a broader data gap in bridging emerging disease modelling and biosurveillance.

## Conclusion

We live in a world where anthropogenic landscape change has reached unprecedented levels^36,37^. Identifying high-risk areas for risk mitigation and management measures that can simultaneously reduce the risk of infectious diseases and preserve biodiversity is highly desirable. We aimed to bridge the gap between infectious disease epidemiology and conservation biology, facilitating a holistic understanding of the spatial scale effects and risks associated with novel infectious disease emergence and paving the way for integrated solutions to protect both human and ecosystem health.

At present, there is no technology that can point out with certainty which infectious diseases will emerge^38^ in these biodiversity-rich and host-rich hotspots, or at what scale they will respond and spread in a landscape. Based on a mechanistic view of the disease emergence process, our gravity models indicate the importance of Jakarta and West Java to epidemic spread and management calculation shows highly managed forests are ubiquitous across the island. Furthermore, we reinforce the power of spatial data and relevance of discussing resolution in spatial planning when quantifying information on the wildlife-livestock-human interface where spillover can be more likely due to forest encroachment. Our results indicate scale testing can be useful to minimise information loss in models for disease emergence risk based on biodiversity forest fragmentation, and spread through human population distribution. Scales coarser than 2000 m present significant information loss, which may mean that coarse scaled models for epidemic and pandemic potential calculations might be of low value for local primary prevention implementation. In addition, this supports the idea that the value of local knowledge is highly relevant for management decisions in conservation actions^39^. Finally, we want to encourage researchers to acknowledge the effects of spatial resolution when dealing with multi-driver processes such as zoonotic risk and biodiversity loss in tropical forests. Our findings are relevant for analysis that can inform primary prevention plans and risk assessments, as the workflow can be adapted to the needs of each forest management category and tailored to mobility data. In practical terms, risk can be coupled with land use planning and prioritisation, and global biodiversity framework targets for 2030, including forest protection, management, and restoration.

## Methods

### Overall workflow

We developed a workflow to estimate epidemic risk from biodiversity loss and land use change. First, we defined the study area, selecting provinces across Java and sourcing key spatial datasets, including forest cover, human population, and administrative boundaries. Next, we identified forest patches and edge areas, applying the species–area relationship (SAR) to estimate microbial diversity for each patch using z-values (e.g., 0.2 or 0.28). We then calculated the estimated risk for novel infectious disease emergence (eRIDE) as the product of patch biodiversity and patch perimeter, generating spatial hazard maps that reflect microbial diversity and fragmentation. To estimate the Population at Risk (PAR), eRIDE values were combined with population density at each pixel and aggregated to both administrative regions and population-based Voronoi polygons. To assess epidemic potential, we applied gravity models incorporating PAR and inter-region distances, comparing outputs from province centroids, Voronoi-based models, and Gaussian decay surfaces. The effects of spatial resolution were evaluated by repeating eRIDE and PAR estimation at resolutions from 100 m to 5 km, with model fit assessed using AICc. Finally, we linked risk estimates to forest management types (e.g., intact, regenerating, agroforestry) and used bivariate mapping and density plots to explore associations between land use practices and emerging disease risk. Further details for each step are below.

### Theoretical framework

The theoretical framework applied here estimates richness values from the species–area relationship (SAR) to connect biodiversity, forest habitat and fragmentation with the risk of exposure to novel infectious diseases^2^. Under this model, habitat division inherently leads to increased exposure to potential sources of harm (hazard)^40^ at the landscape-level. The hazard is posed by the entire range of disease-causing microbial diversity within a habitat, rather than specific traits of individual disease-causing agents. The framework is based on assumptions that generalise disease behaviour allowing it to be modelled in a mechanistic fashion^2^. We provide a summary of biodiversity and edge amount in a biodiverse region of the world as a starting point for quantifying the amount of human-nature interface per area. We evaluate data by first estimating patch biodiversity and the risk of emerging infectious disease emergence.

### Biodiversity model

The number of mammalian viruses for zoonotic infections proportionately increases with mammalian species richness (Fig. S2). Based on this assumption, we build a microbial diversity model that is used to estimate the risk for novel infectious disease emergence (eRIDE). Increasing contact due to human encroachment and landscape fragmentation with corresponding species diversity decline are likely to act antagonistically to affect hazards from novel pathogens. We calculate eRIDE^2^ using the measures of biological diversity derived from landscape fragmentation and decline rates based on species-area relationship from literature. From every patch, a measure of alpha microbial diversity is measured. eRIDE is a ‘bottom-up’ estimate based on all biodiversity from forest patch and edge metrics and species-area rules. The species-area relationship model here uses a power-law relationship. We derived data from species-habitat-area relationships expected for microbial communities and vertebrates (z-value = 0.23 ± 0.13, approximated to 0.20, and z-value = 0.28, Table S2), considering forests as habitats in a binary forest-non-forest space. Rate of species decline was set as 0.2 based on island biogeographic empirical estimates at a conservative lower level. Because *z* varies according to matrix type (as the intervening matrix becomes more permeable, the *z*-value decreases), we expect z-values in land to be smaller as the terrestrial matrix is not expected to be completely impermeable to terrestrial wildlife as the ocean. Because of that, we use edges and moving windows to evaluate spillover risk. Regardless, we also run our estimates for z-value = 0.28 and compare them with z = 0.20 in order to check the sensitivity of the trends observed at optimal spatial resolution values for eRIDE. The variation encompasses empirically derived values for vertebrate species assemblages across different habitat patches present in different types of landscapes and island systems (Table S3, extracted from Matthews et al.^41^).

The land use layer was upscaled from the native resolution of 30 m to 100 m values in spatial resolution to match the population data through nearest neighbor resampling. After that, we repeated our workflow for progressively coarser spatial scales.

Within our system, all approximations of total microbial species diversity can be relative measures on an arbitrary scale. We assume that our habitat patches are not linked following division, but our estimates incorporate the dense tree cover areas as forest habitat. The overall risk coming from any pathogen is based on the biodiversity model and interactions with forest edges. We assume that microbial species-rich areas inland vary as a function of patch area and empirical number of species of birds and mammals, whereas patches considered as habitats are classified as forest from land cover data.

Habitat fragmentation was based on forest cover extracted from GLAD GlobeCover for 2019^22^ using landsat images (Fig. S3). The mosaic images were processed at 0.00025 dd resolution (~ 30 m). All forest land cover classes (landclass codes from 50 through 116) were considered as habitat. We adapted the original algorithms from Wilkinson et al. (2018)^2^ to R 4.5.0^26^ and GRASS GIS 8.4^42^ through custom codes based on the *lsmetrics* package workflow^43^.

### Estimated risk of infectious disease emergence

The risk of infection emergence from forests into the expanding human population (*R*), was considered as the product of the relative number of potential disease-causing agents, which we assume to scale linearly with forest patch (_*i*_) microbial diversity (*S*_*i*_)^44^, and the area over which the population first comes into uniform contact with this habitat, which we assume is represented by the perimeter of the habitat fragment (*P*_*i*_). So, hazard is proportional to patch biodiversity, whereas we have that:

$$R_{i} = S_{i} P_{i}$$

And total risk *R* is

$$R = \sum\limits_{i}^{{}} {R_{i} }$$

We assume eRIDE correlates with a possible hazard. Our gravity models take into account that the estimated hazard reflects a higher risk of human exposure to newly emerging pathogens. We assume that disease spread between nearby areas is proportional to the product of their population densities, making routes with high population density more likely pathways for spread^2^. Human population at risk (PAR) is defined as the product of the pixel eRIDE index and the human population at that location. That is the basis for the flow calculation for the target subregions applying three versions of a gravity model^2,45^. The epidemic potential map from eRIDE was built from gravity to terrestrial areas of Java. Then we calculate population at risk estimates and epidemic potential across the following administrative regions: Bali Province, Banten Province, Jakarta Raya Special District, Jawa Barat (West Java) Province, Jawa Tenga (Central Java) Province, Jawa Timur (East Java) Province, and Yogyakarta Special region. We used WorldPop population data^46^ and administrative boundaries *rnaturalearth* data^47^. We used the human population at ~ 100 m resolution as the total number of people per grid-cell based on the 2020 population census^48^ to infer the effects from a potential outbreak through direct transmission using gravity models and a pixel-based cumulative potential surface for PAR^49^, representing the given risk of novel emerging zoonoses. The estimated PAR was defined as the product of the eRIDE value and the population at a given location.

### Modelling the impact of upscaling

After calculating eRIDE and PAR, we developed models to understand how upscaling working resolution influences statistics for eRIDE and PAR. In previous work, our eRIDE metric was a considerably better predictor of Ebola virus disease (EVD) emergence in Africa compared to other habitat fragmentation metrics, which were individually poor predictors for a wide range of scales (from 300 m up to 60 km)^2^. Within 5 km of outbreaks, eRIDE was 10–12-fold higher than background values and 7–eightfold increase in areas within 5–60 km of known EVD outbreak index cases. Our main question here was to understand how much resolution matters in terms of information loss (variation, SD) and generality (averages maintained) for analyses using this eRIDE modelling framework. Resolution changes are viewed in terms of computational cost (number of pixels to process) and represent the upscaling effect. Predictor data for costs was extracted for the following resolutions (N = 18): 100, 150, 200, 250, 300, 350, 400, 500, 600, 700, 800, 900, 1000, 2000, 2500, 3000, 4000, and 5000 m. Information loss —the degree to which information at a coarser scale deviates from the observed values at a finer scale of spatial measurement—was modelled as the difference in SD values from the reference resolution (100 m) for a given metric. Model plausibility was compared through the corrected Akaike Information Criteria. A no-effect, intercept-only model was competed with all concurrent models including general linear, piecewise regression^50^, and non-linear additive models with limited basis dimension for the smooth term (*k*) up to four. Models with ΔAICc were considered the most plausible models. Weight of evidence was inspected to determine the model that stands out as the most appropriate representation of the underlying processes.

### Epidemic risk models

We apply gravity models based on human population at risk for our study region using two area division types and discuss epidemic potential. To estimate the potential for epidemics from an emerging disease, we modelled disease spread across target provinces of Indonesia (Admin-1 states provinces): Banten, Jakarta Raya, West Java, Central Java, Yogyakarta special region, East Java, and Bali. Our models assume that the risk flow follows an inverse-linear relationship with the distance between two population centres, aligning with the principle that proximity facilitates interaction flow^45^. The denominator exponent and scaling constant were set to one. We assume the potential for spread between any two areas was proportional to the product of the population, so that epidemics were likely to travel along paths of high population density. We applied three methods for the calculation of epidemic risk. First, we calculate a gravity model using the provinces of Java. This method ignores islands in terms of their spatial information but takes into account the PAR for the entire province, including the PAR in islands for a given province. It considers the centroid of each province as the base for calculation of distances in the gravity model. In the case of Java, most provinces are composed of small islands around their respective majoritarian area in Java main island. In order to population and add anisotropy here in increasing complexity, we calculate risk using two more methods.

The second method models high-level population-Voronoi-tessellation areas forced upon identified provinces and then calculates a gravity model based majority occupied in these areas (Fig. S5). When constructing a gravity model, particularly for epidemic risk assessment, using Voronoi polygons based on high population values provides a more realistic representation of population distribution compared to simply relying on province boundaries and their centroids. While centroids represent the geometric center of a province, they fail to capture the true spatial variation in population density, especially when the distribution is uneven across the region. Voronoi polygons, derived from high-density population centers, offer a better approximation of how populations are spatially distributed, or the spatial domains of a region based on where most people are present (here, using the 95th population quantile, N = 100). Although not perfectly accurate, these polygons capture the influence of densely populated centers, which is crucial in gravity models that rely on spatial interactions, such as distance and neighborhood. In addition to adding more realism to the analysis, Voronoi polygons remain computationally efficient, unlike pixel-based gravity models, which are more data-intensive. This approach strikes a balance between improving accuracy and maintaining manageable computational demands, making it suitable for large-scale applications. Moreover, by forcing the Voronoi coverage to align with province boundaries (i.e., each Voronoi polygon is assigned to the province where the majority of its area is located), each polygon retains the administrative boundary information (Fig. S5). This feature makes Voronoi polygons not only ideal for large-scale epidemic risk assessments but also useful for more local spatial planning tasks that require adherence to administrative limits. Province-level governance actions—such as public health interventions, infrastructure planning, or resource allocation—can be aligned with these high-population domains for each region, ensuring that political limits are respected while containing meaningful information on population centers, given and received risk.

Lastly, we used a cumulative surface based on pixel-level contiguity and 100 km window for Gaussian decay of estimates as a proxy for given risk. This distance surpasses the average commuting length in the largest Indonesian metropolitan areas (~ 22 km), allowing for the reach of rural areas and neighbouring provinces considering the distances covered by motorized vehicles^51^. To assess the potential of each source area to contribute to an epidemic, we rank the total estimated flow for every area and their contributions to risk in all other regions of Java. So the received epidemic risk is later calculated for each province considering the province source of given risk for both all vector-based calculations applied. No outbreak area was used to validate epidemic potential estimates, because current observations of emerging disease data is proven to be heavily determined by detection^10^.

### The relationship between risk and forest management/primary prevention targets

Finally, we extracted values for populations at risk to highlight regions of interest that are under varying forest management regimes, which can be viewed as where primary prevention actions— such as pathogen spillover surveillance, conservation actions, deforestation reduction and sustainable practices—may take place. We evaluate no management/mostly intact, low-management/regenerating and managed/ agroforestry areas and their land cover intensity across Java island at different levels of PAR. We believe this analysis helps understand how land management practices may increase infectious disease emergence risk. The source for our forest management layer was Lesiv et al.^52^ with reference data at 100 m (Table S4). All spatial data was warped to the Pseudo-Mercator (EPSG:3857) projected coordinate system and the World Geodetic System 1984 datum. We build bivariate maps using the quantiles for management cover and PAR using the provinces and population-informed Voronoi areas as spatial units. We finally inspect data density distribution of land cover proportions to unravel the association patterns between forest management and high levels of PAR divided as quantiles.

## Supplementary Information

Below is the link to the electronic supplementary material.

Supplementary Material 1

## Data Availability

All datasets used in our workflow are openly available online, including land cover data (https://glad.umd.edu/dataset/global-land-cover-land-use-v1), forest management data (https://zenodo.org/records/4541513), Virion atlas on vertebrate host viruses (https://zenodo.org/records/5819056)^53^, host species ranges from the IUCN Red List (https://www.iucnredlist.org/), administrative boundaries (https://cran.r-project.org/web/packages/rnaturalearthdata/index.html)^47^, and human population data (https://hub.worldpop.org/geodata/summary?id=6376)^46^.

## Code availability

The code used for the analyses is available on GitHub (https://github.com/renatamuy/eride) and Zenodo (10.5281/zenodo.8397517). An example script demonstrating the analysis workflow can be accessed here: 02_eride_toy.R.
