# Supplementary material for: Upscaling effects on infectious disease emergence risk emphasize the need for local planning in primary prevention within biodiversity hotspots
Source: Sci Rep. 2025 Oct 27;15:37504. doi: 10.1038/s41598-025-21514-4 (PMC12559317; doi:10.1038/s41598-025-21514-4)
Supplement: Supplementary file 1 — Supplementary Material 1 [file 41598_2025_21514_MOESM1_ESM.docx]

## **Supplemental Material**

####

**Upscaling effects on infectious disease emergence risk emphasize the need for local planning in primary prevention within biodiversity hotspots**

[*Renata L. Muylaert*](https://orcid.org/0000-0002-6466-6210)*^1*^,* [*David A. Wilkinson*](https://orcid.org/0000-0002-9986-6212)*^2^, Evita Izza Dwiyanti^3^,* [*David T. S. Hayman*](https://orcid.org/0000-0003-0087-3015)*^4^*

*^1^ Sydney School of Veterinary Science, Faculty of Science, The University of Sydney, Sydney, 2050 New South Wales, Australia.*

*^2^ UMR ASTRE, CIRAD, INRAE, Université de Montpellier, Plateforme Technologique CYROI, Sainte-Clotilde, Reunion, France*

*^3^ Alam Sehat Lestari, Jakarta, Indonesia*

*^4^ Molecular Epidemiology and Public Health Laboratory, Hopkirk Research Institute, Massey University, Palmerston North, New Zealand*

* Corresponding author**:** renata.muylaert@sydney.edu.au

####

#### **This file includes:**

Tables S1-3

Figures S1-9

**Table S1.** Variation in Average values for spatial variables considering different Z-values. The effect of coefficient variation on average microbial diversity, eRIDE and PAR estimates is displayed.

**z components value**

z = 0.20 bio 5.26

z = 0.28 bio 10.77

z = 0.20 eRIDE 2.42

z = 0.28 eRIDE 4.3

z = 0.20 PAR 16.11

z = 0.28 PAR 26.25

#####

##### **Table S2. Mean and standard deviations of empirically estimated *z*-values (rate of decline*)* for the SAR relationship**. Values are calculated from island species–area relationship (ISAR) and RANSAC versions of each dataset. Reference value for estimated risk for novel infectious disease emergence (eRIDE) calculation was set as 0.20 and 0.30.

| **Model** | **z-value** | **Reference** |
| --- | --- | --- |
| Invertebrates (n=20) | 0.24 ± 0.21 | (Matthews *et al.*, 2016) |
| Plants (n=8) | 0.14 ± 0.09 | (Matthews *et al.*, 2016) |
| Vertebrates (n=69) | 0.23 ± 0.13 | (Matthews *et al.*, 2016) |
| Microbial communities (n=1) | 0.28 | (Bell *et al.*, 2005) |

**Table S3.** List of z-value estimates for vertebrates from different island groups [(Matthews et al. 2019)](https://paperpile.com/c/Vv4AtK/Dlit) ordered alphabetically and by group (Birds, Mammals, Herpetofauna).

| Taxon | Archipelago | z |
| --- | --- | --- |
| Birds | French Frigate Shoals atoll and Perl & Hermes Reef (S.W.  Hawaii) | 0.54 |
| Birds | Maine, USA | 0.53 |
| Birds | Åland Islands, S.W. Finland | 0.51 |
| Birds | Boston Harbor Islands, Massachusetts | 0.5 |
| Birds | Indian Ocean Islands | 0.48 |
| Birds | Georgian Bay Islands, Great Lakes, USA | 0.47 |
| Birds | Fathom Five Islands, Great Lakes USA | 0.4 |
| Birds | Michigan Lake, Great Lakes, North America | 0.39 |
| Birds | New Zealand islands | 0.31 |
| Birds | Dahlak Archipelago, Red Sea | 0.31 |
| Birds | Samoa offshore Islands, Pacific | 0.25 |
| Birds | Hawaiian Islands, Pacific | 0.24 |
| Birds | Lago Guri islands, Venezuela | 0.24 |
| Birds | Philippine Islands | 0.24 |
| Birds | Lesser Antilles, Caribbean Sea | 0.21 |
| Birds | West Indies, Caribbean Sea | 0.19 |
| Birds | New Zealand islands | 0.17 |
| Birds | St. Lawrence, Great Lakes, USA | 0.17 |
| Birds | Cape Verde, Atlantic | 0.15 |
| Birds | Vanuatu, Pacific | 0.13 |
| Birds | Vava'u Group, Tonga | 0.07 |
| Birds (forest birds) | Andaman islands, Indian Ocean | 0.18 |
| Birds (passeriformes) | Thousand Island Lake Region, China | 0.21 |
| Herpetofauna | Green Bay, Lake Michigan USA | 0.46 |
| Herpetofauna | Western Lake Erie, USA | 0.42 |
| Herpetofauna | Gulf of California, Central America | 0.34 |
| Herpetofauna | St. Lawrence, Great Lakes, USA | 0.33 |
| Herpetofauna | Georgian Bay Islands, Great Lakes, USA | 0.31 |
| herpetofauna | Grenadines Islands, Caribbean Sea | 0.27 |
| Herpetofauna | Mediterranean Islands | 0.23 |
| Herpetofauna | Lesser Antilles, Caribbean Sea | 0.16 |
| Herpetofauna (amphibians) | Zhoushan archipelago, China | 0.31 |
| Herpetofauna (reptiles) | Adriatic Sea islands | 0.36 |
| Herpetofauna (reptiles) | Aegean Sea, Greece | 0.28 |
| Mammals | Green Bay, Lake Michigan USA | 0.41 |
| Mammals | Gulf of Maine, North America | 0.4 |
| Mammals | Papua New Guinea Islands | 0.35 |
| Mammals | Georgian Bay Islands, Great Lakes, USA | 0.35 |
| Mammals | Great Bay, Japanese Sea | 0.31 |
| Mammals | Adriatic Sea islands | 0.29 |
| Mammals | Fathom Five Islands, Great Lakes USA | 0.26 |
| Mammals | Japanese archipelago | 0.23 |
| Mammals | Gulf of Maine, North America | 0.21 |
| Mammals | Alexander Archipelago, Alaska | 0.18 |
| Mammals | New England, MA, USA | 0.06 |
| Mammals (bats) | West Indies, Caribbean Sea | 0.19 |
| Mammals (bats) | Baja, California | 0.09 |
| Mammals (non-volant) | West Indies, Caribbean Sea | 0.21 |
| Mammals (primates) | SE Asian Islands | 0.21 |
| Mammals (shrews) | Sysmä Lake, Finland | 0.27 |

##### **Table S4. Management level types and their respective pixel code.** The description was adapted from Lesiv et al [^47^](https://paperpile.com/c/jkfey0/ALH1). This forest management layer presents 100 m resolution data for 2015.

| **Pixel code** | **Type Broad** | **Type Specific** | **Description** |
| --- | --- | --- | --- |
| 11 | No management | No management | Not disturbed at 100 m buffer but can contain low human impact as houses and small crops within a 500 m buffer. |
| 20 | Managed | Managed low-level | Naturally regenerating forests with signs of forest selective logging, clear cuts etc. |
| 31 | Managed | Managed long time | Planted forests with more 15 year rotation time. |
| 32 | Managed | Managed short time | Planted forests with 15 year rotation time or less. |
| 40 | Managed | Managed oil Palm | Oil palm plantations. |
| 53 | Managed | Managed agroforestry | Orchards, olives, apples, nuts, cocoa, mixed crops, shifting cultivation, trees in built up areas. |


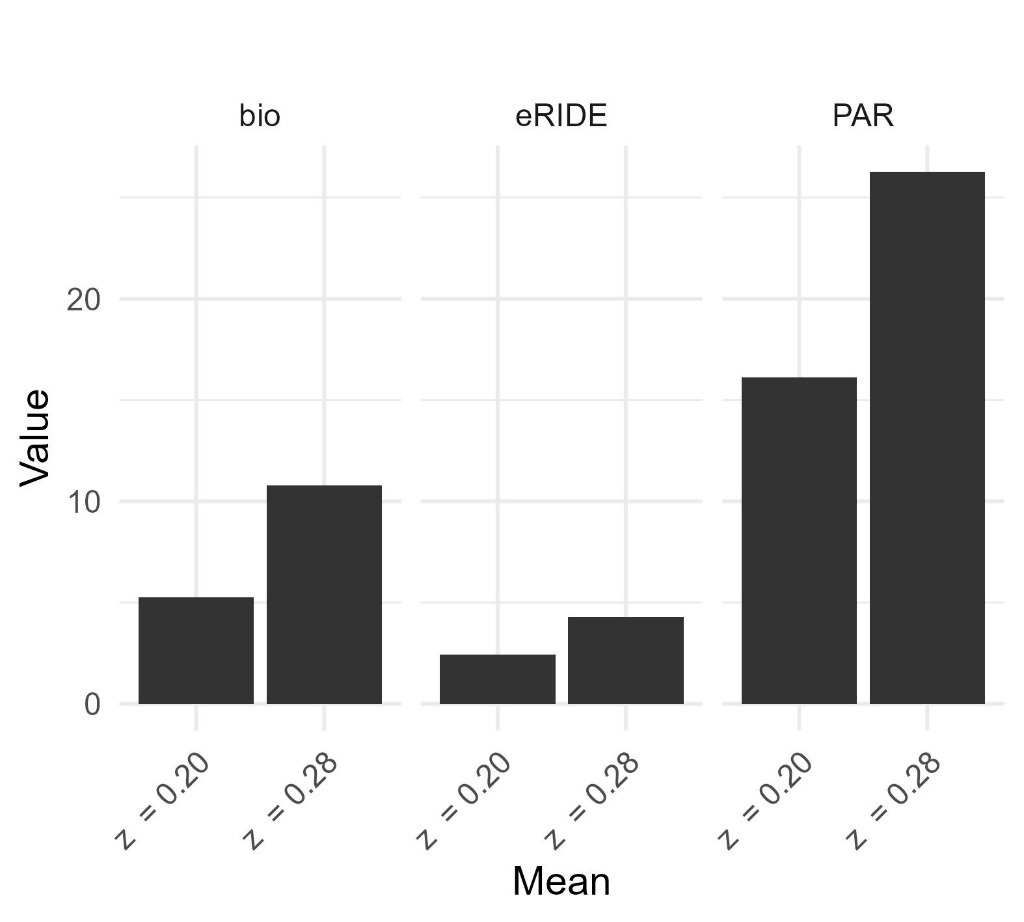


**Figure S1. Comparison of estimates for different z-values for the species-area relationship (SAR) at a 500 m scale.** The effect of coefficient variation on microbial diversity, eRIDE and PAR estimates is displayed.

#####


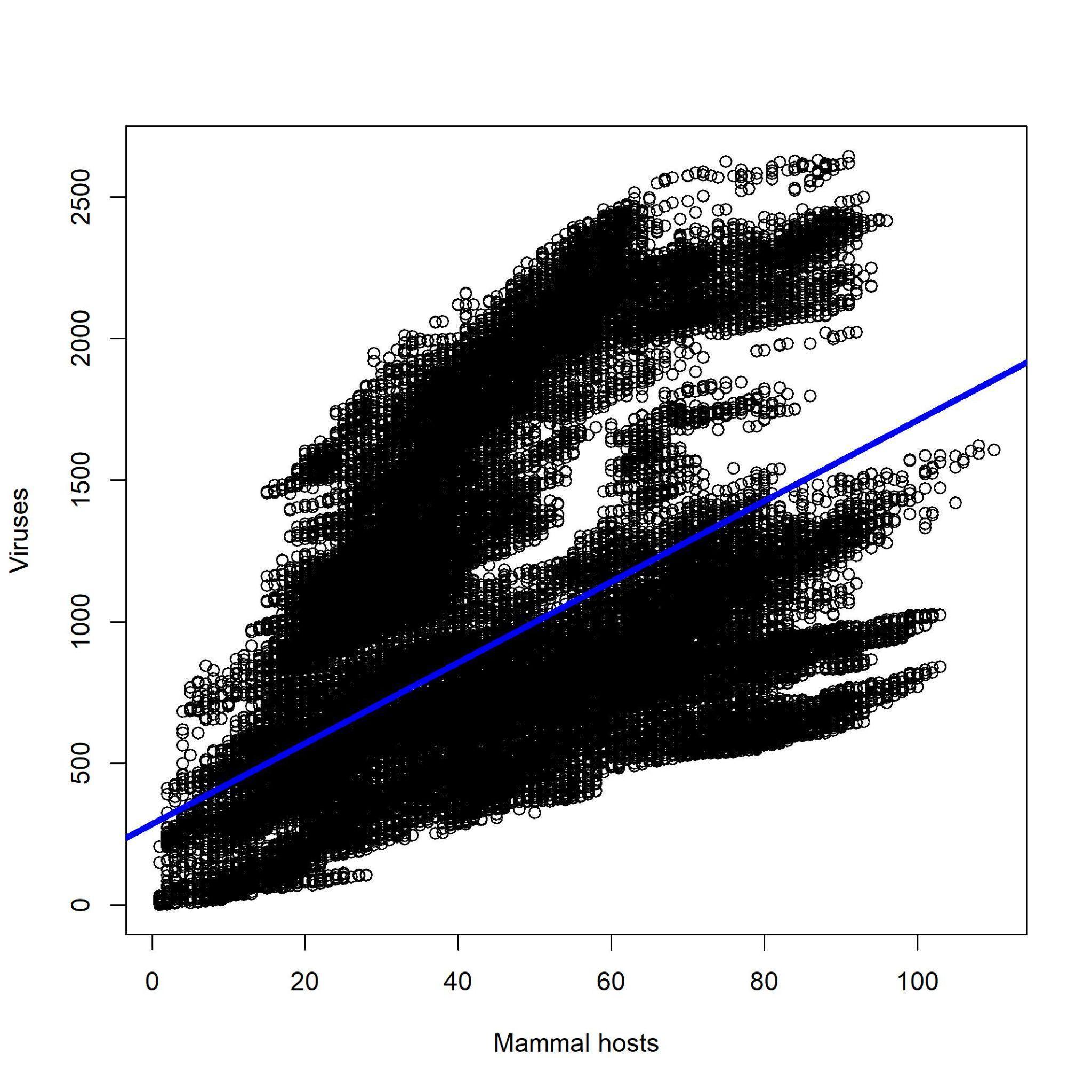


##### **Figure S2. Positive relationship between terrestrial mammals species richness and number of mammal viruses displayed with a simple linear model.** Hosts of viruses were extracted based on infection data and their distribution accessed on 2nd Feb 2023 in Virion v0.2.1 ^1^ and IUCN range data ^2^ at 0.25 dd resolution (~27 km). For our posterior analysis, we used total estimated species richness to calculate the estimated risk for novel infectious disease emergence (eRIDE), since pathogen diversity scales up with total diversity.


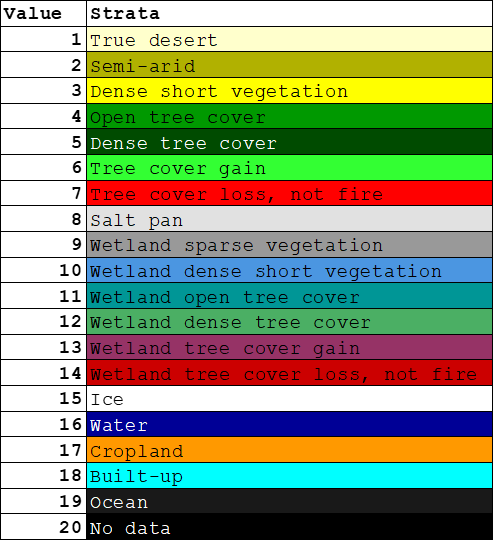


##### **Figure S3. Legend of land cover raster used to calculate estimated risk for novel infectious disease emergence (eRIDE).** Forest codes used: 5, 6, 12, 13, which are dense tree cover, tree cover gain, wetland dense tree cover and wetland tree cover gain. Data source: GLAD GlobeCover for the year 2019 [^20^](https://paperpile.com/c/jkfey0/crOx) (<https://glad.umd.edu/dataset/global-land-cover-land-use-v1>)


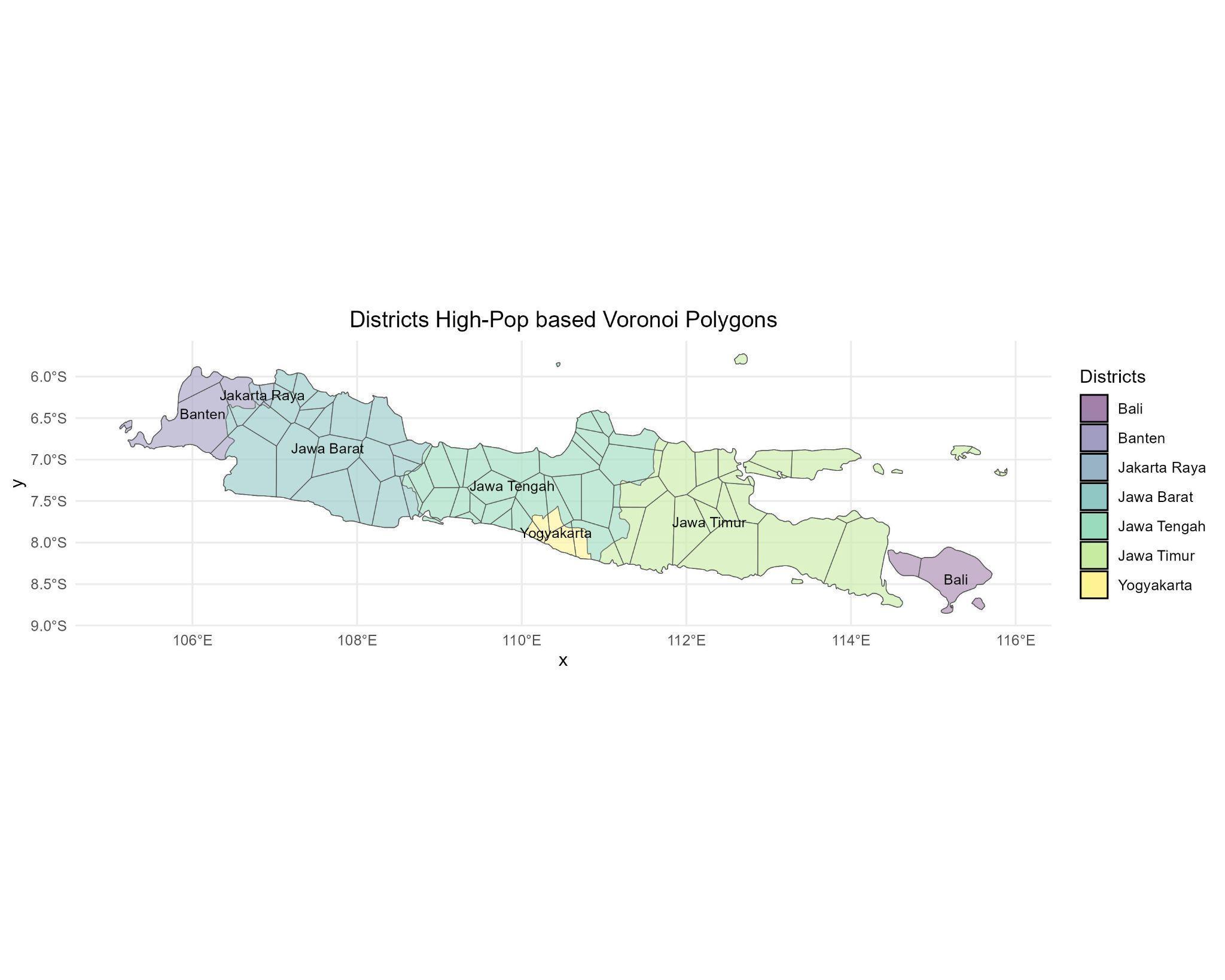


##### **Figure S4. High-population spatial domains through Voronoi Tessellation.** The Voronoi polygons were linked to original province extents based on a majority of area rule. We used three methods for receiving risk estimates**:** administrative province gravity model, high-population spatial domains through Voronoi Tessellation (displayed as map polygons) gravity model and pixel-based analysis.


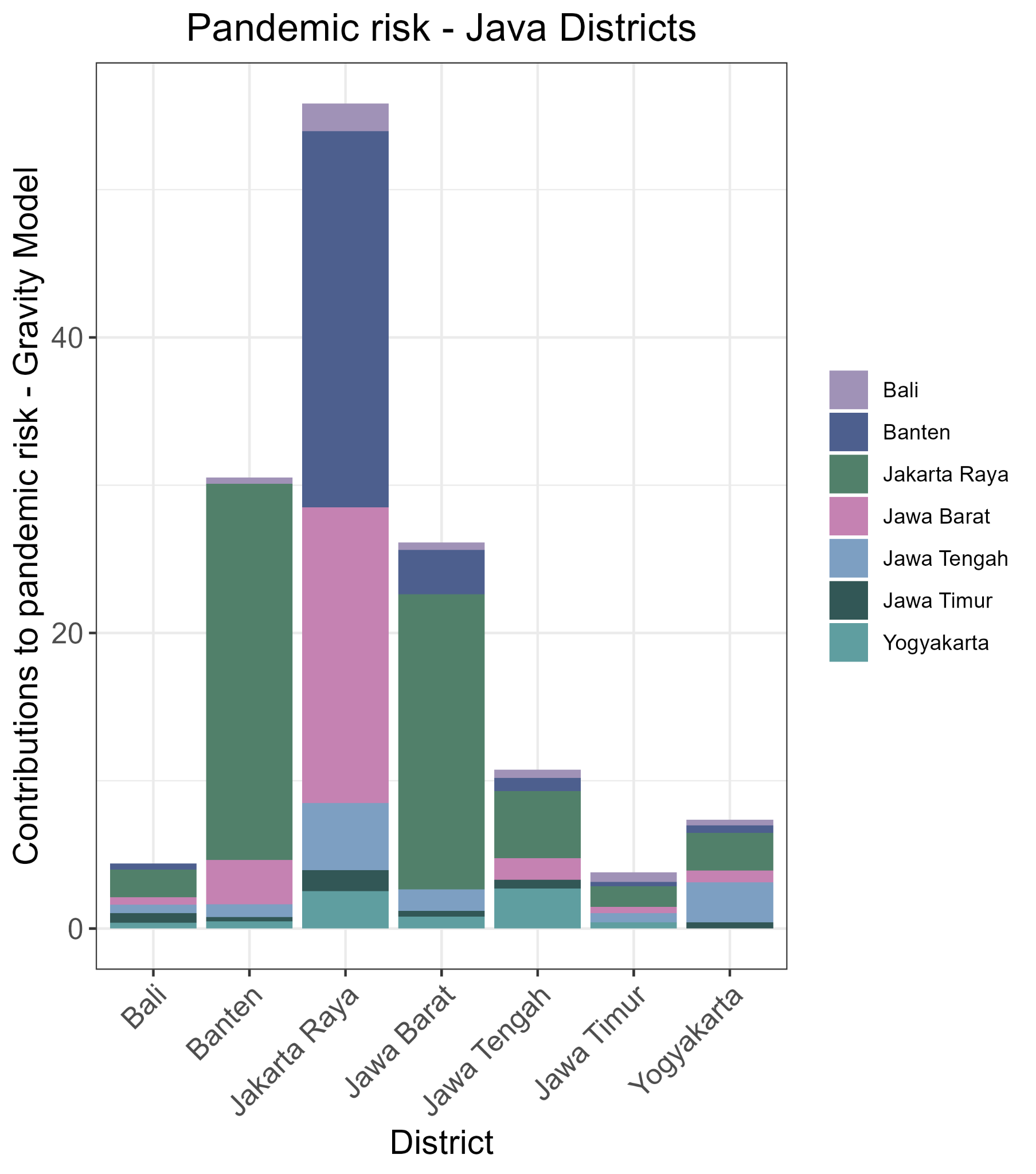


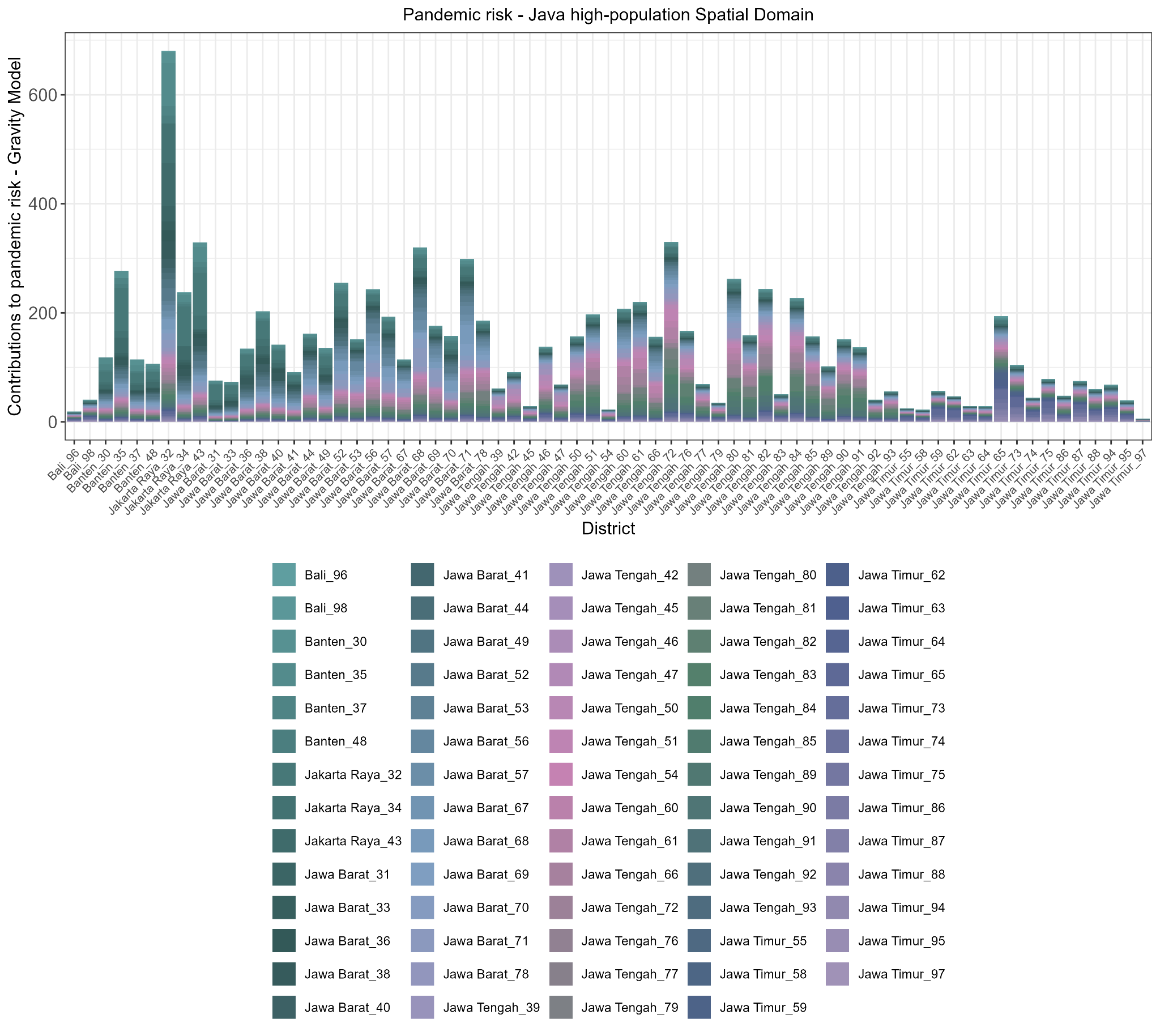


**Figure S5. Epidemic risk values and zones of influence using the gravity model.** The overall received epidemic risk based on estimated risk for novel infectious disease emergence (eRIDE) and PAR was calculated for each (A) region and (B) high-populated Voronoi spatial domains (native resolution of 100 m for the population data used as input).

##### **
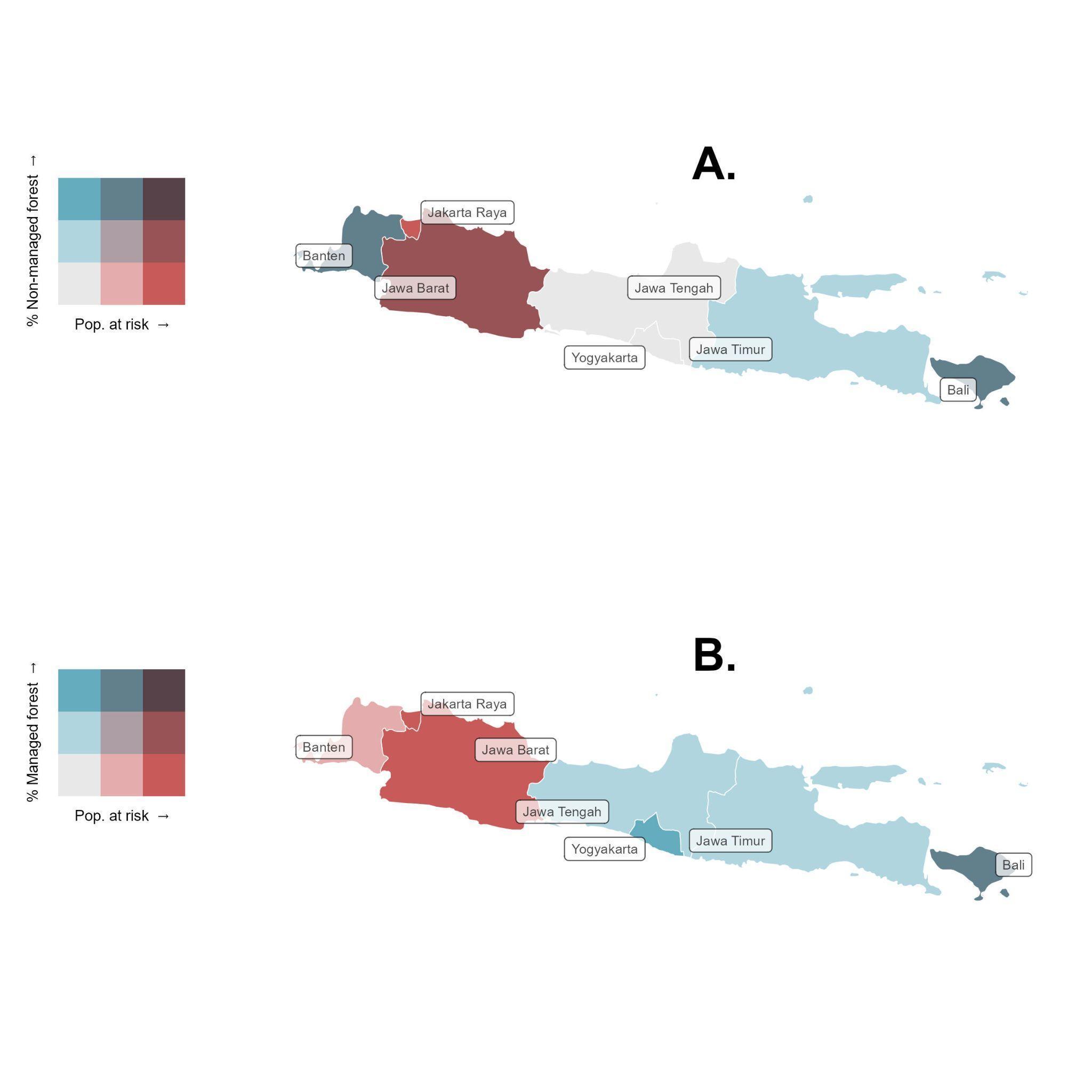
Figure S6. Area covered by non-managed forest (A) and (B) intensely managed agroforestry and the distribution of population at risk based on gravity models throughout provinces of Java.**


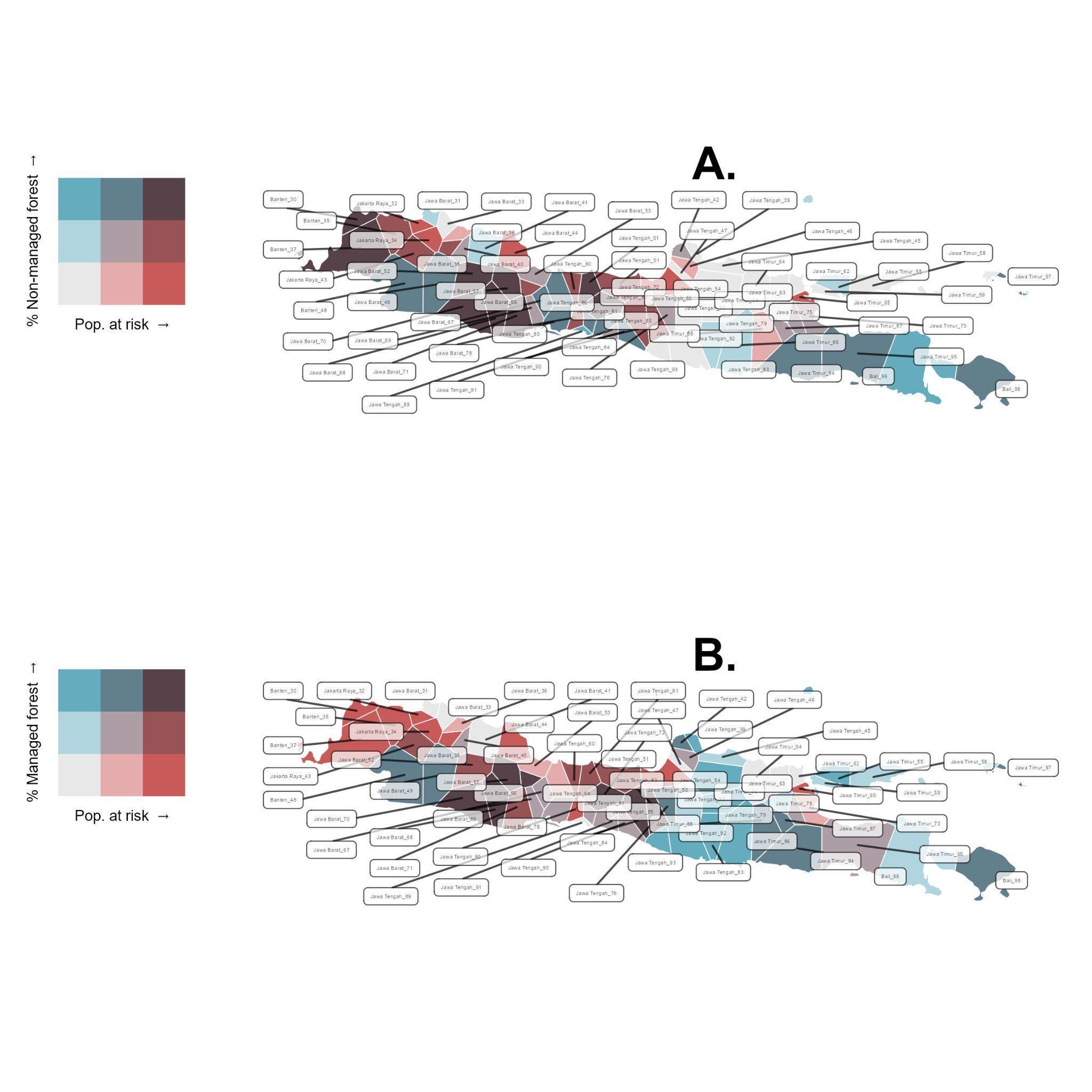


##### **Figure S7. Area covered by non-managed forest (A) and (B) intensely managed agroforestry and the distribution of population at risk.** Data for population at risk was calculated using the Voronoi-tessellation of highly populated centres and gravity models throughout provinces of Java.


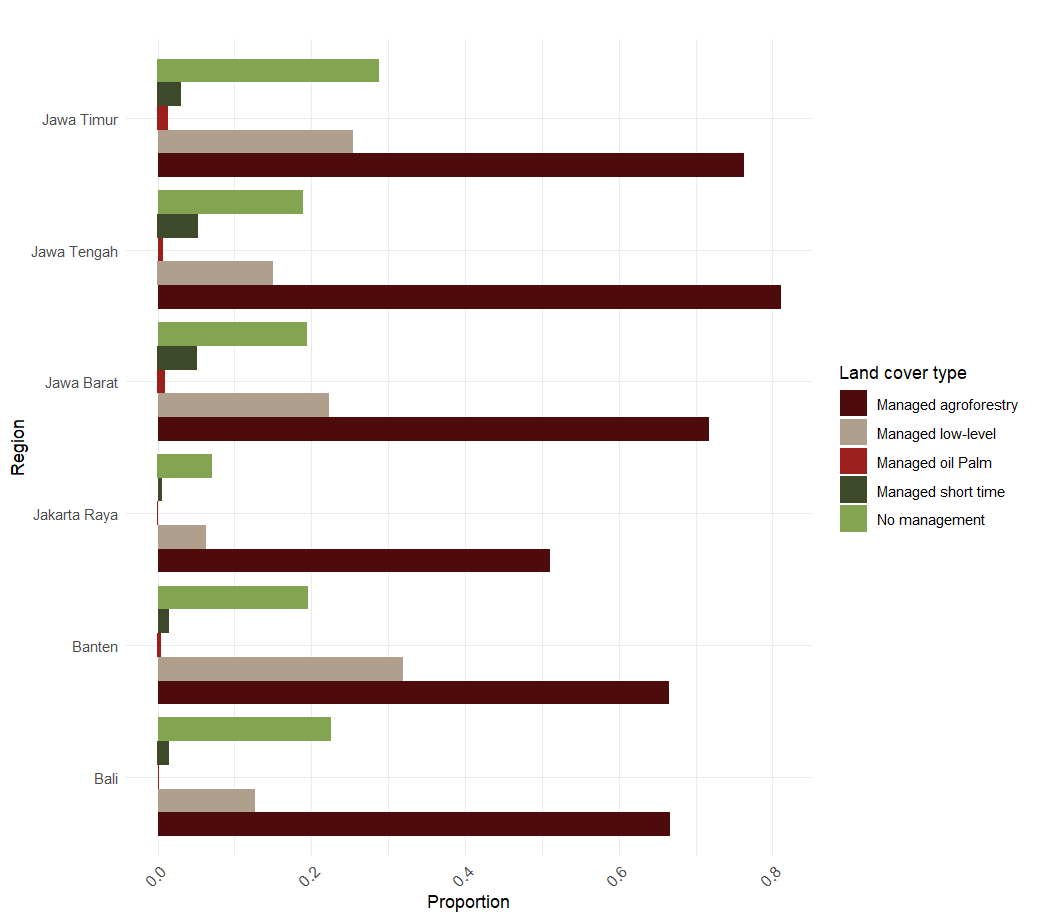


##### **Figure S8. Categories of managed and unmanaged forest cover values and distribution in Java provinces.**


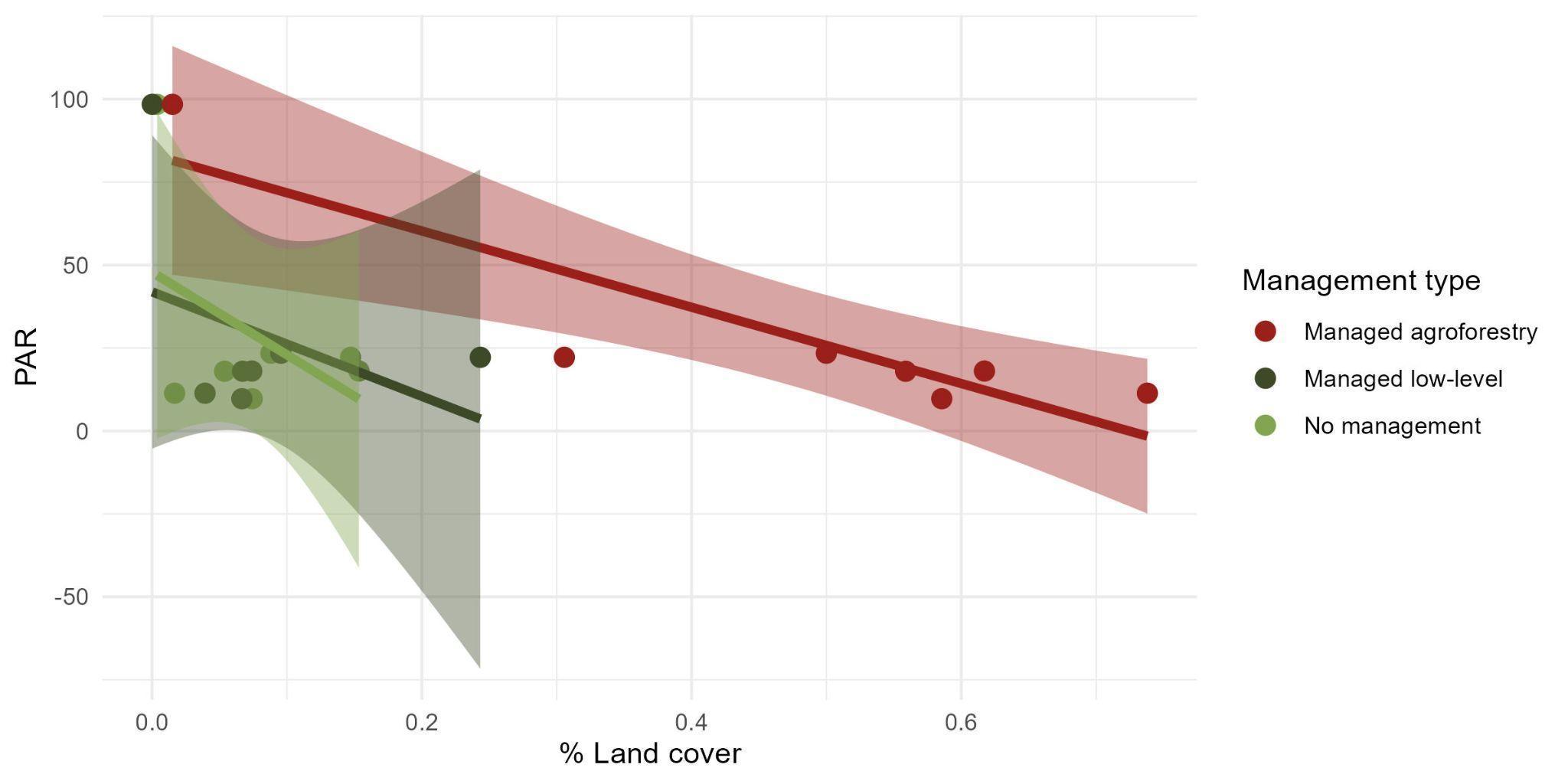


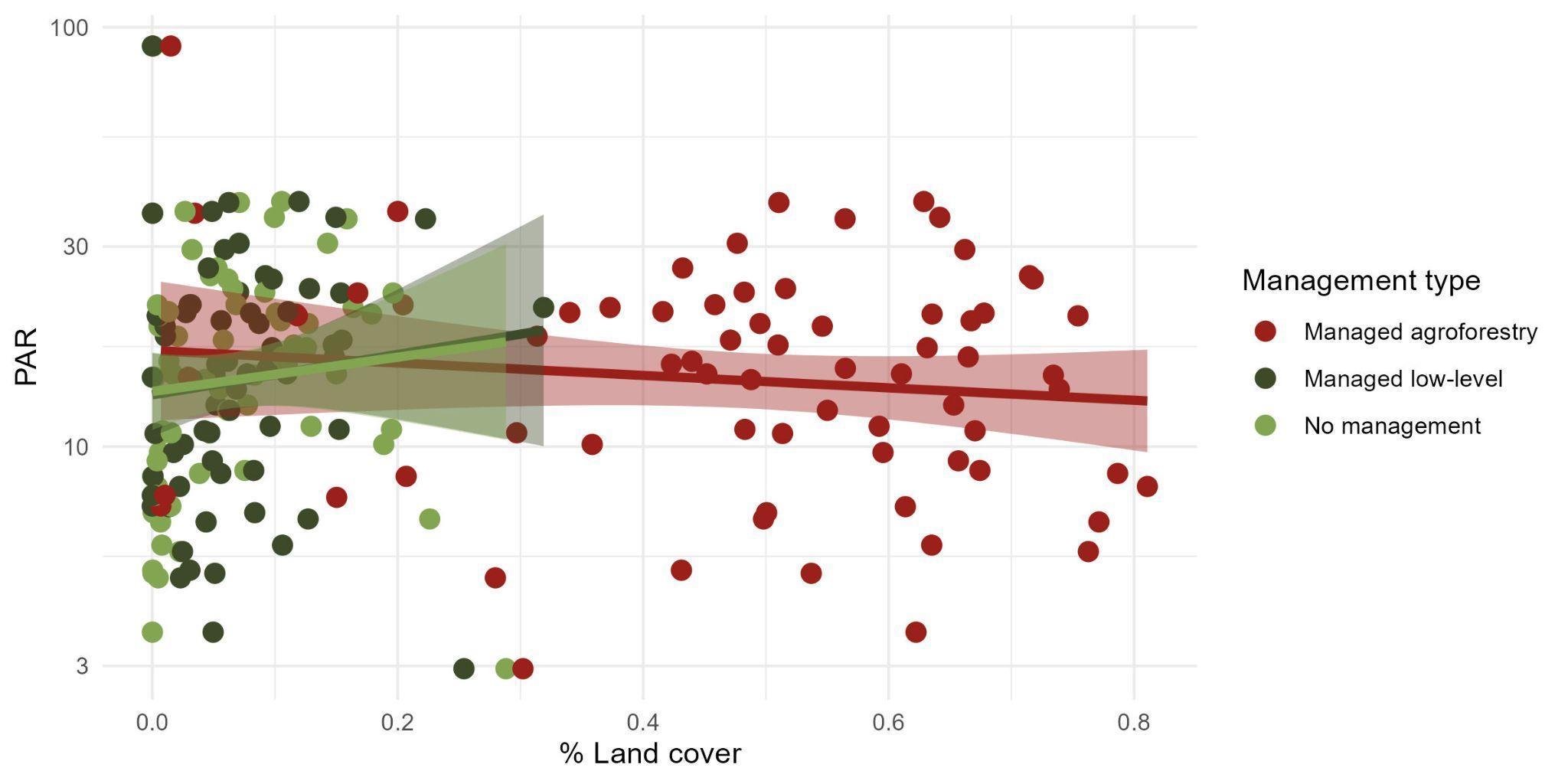


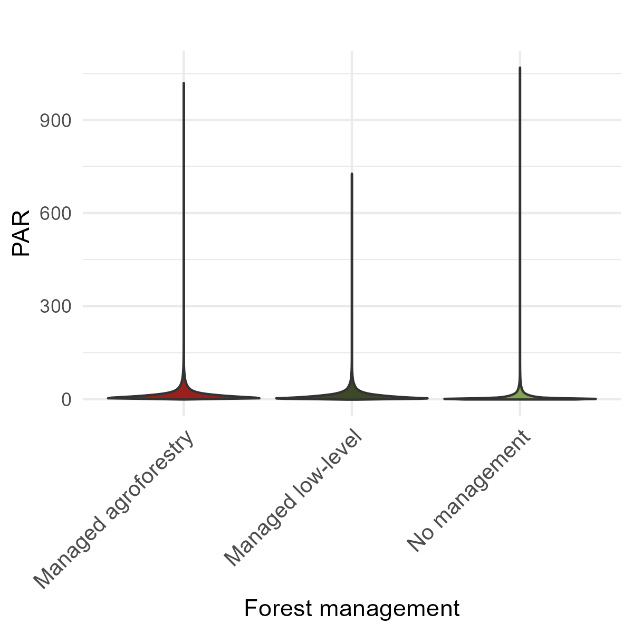


**Figure S9. Relationship between land cover of different forest management types across provinces and population at risk estimates from eRIDE model (100 m).** Top panel: province-level data aggregation. Middle panel: high-pop driven gravity model spatial domains. Top and middle panel display a linear function on the top of the data with 95% confidence intervals. and Bottom panel: violin plot of pixel-level data across Java.

**References**

1. Carlson, C. J. et al. The Global Virome in One Network (VIRION): an Atlas of Vertebrate-Virus Associations. MBio e0298521 (2022).
2. IUCN. The IUCN Red List of Threatened Species. Version 2020-2. The IUCN Red List of Threatened Species. Version 2020-2. https://www.iucnredlist.org. Downloaded on 09 May (2020).
